# Gestational diabetes induces behavioral and brain gene transcription dysregulation in adult offspring

**DOI:** 10.1038/s41398-020-01096-7

**Published:** 2020-11-25

**Authors:** Keren Aviel-Shekler, Yara Hamshawi, Worood Sirhan, Dmitriy Getselter, Kolluru D. Srikanth, Assaf Malka, Ron Piran, Evan Elliott

**Affiliations:** Azrieli Faculty of Medicine, Bar Ilan University, Safed, Israel

**Keywords:** Molecular neuroscience, Autism spectrum disorders, Epigenetics in the nervous system

## Abstract

The etiology of Autism Spectrum Disorders (ASD) includes a strong genetic component and a complicated environmental component. Recent evidence indicates that maternal diabetes, including gestational diabetes, is associated with an increased prevalence of ASD. While previous studies have looked into possible roles for maternal diabetes in neurodevelopment, there are few studies into how gestational diabetes, with no previous diabetic or metabolic phenotype, may affect neurodevelopment. In this study, we have specifically induced gestational diabetes in mice, followed by behavioral and molecular phenotyping of the mice offspring. Pregnant mice were injected with STZ a day after initiation of pregnancy. Glucose levels increased to diabetic levels between E7 and E14 in pregnancy in a subset of the pregnant animals. Male offspring of Gestational Diabetic mothers displayed increased repetitive behaviors with no dysregulation in the three-chambered social interaction test. RNA-seq analysis revealed a dysregulation in genes related to forebrain development in the frontal cortex and a dysregulation of a network of neurodevelopment and immune related genes in the striatum. Together, these results give evidence that gestational diabetes can induce changes in adulthood behavior and gene transcription in the brain.

## Introduction

Autism Spectrum Disorder (ASD) is a neurodevelopmental and behavioral disorder defined by deficits in social interaction and communication and presence of repetitive behavior^1^. ASD is associated with a variety of prenatal, perinatal and postnatal etiologies, and its current prevalence is above 1% of the children born in this decade^2^. In addition, ASD is more likely to appear in males than females by a factor of 4:1.

The etiology of autism includes a strong genetic basis. Specific CNVs, de novo mutations, and cumulative effects of SNPs have all been implicated in the genetic etiology of ASD. In addition, there are specific genetic syndromes that include the autistic phenotype, including fragile X syndrome, Timothy syndrome, and tuberous sclerosis^3,4^. In addition to the genetic etiology, epidemiological studies have suggested that there are environmental risk factors that are associated with increased ASD prevalence, and may contribute to the development of ASD. In general, prenatal environmental factors often induce neurodevelopmental disorders, such as in fetal alcohol syndrome^5^. In the context of autism, recent evidence has suggested that risk factors for the development of ASD include activation of the maternal immune system or usage of particular medications during pregnancy^3^.

Several epidemiological studies have shown that there is an association between metabolic conditions, including diabetes, during pregnancy and ASD^6–9^. Interestingly, not only is preexisting diabetes associated with autism, but also studies have found an association between autism and gestational diabetes mellitus (GDM). A large epidemiological study found that maternal diabetes, especially GDM, is associated with an increased risk of ASD in offspring^7^. Furthermore, a meta-analysis of several studies has verified an association between GDM and the incidence of ASD in the offspring^10^. In addition, GDM may be associated with a more wide range of neurodevelopmental disorders, particularly Attention Deficit Hyperactivity Disorder^11^. These studies suggest that even the acute effects of gestational diabetes may have an effect on neurodevelopment and behavior.

GDM is a condition that appears during pregnancy in woman with no signs for diabetes of any type before the pregnancy. Approximately 7% of pregnant woman are diagnosed with GDM^12^. In GDM, blood glucose levels are elevated to inappropriately high levels. Most of those women go back to healthy glucose levels after parturition. For the health of the mother and the fetus, the maintenance of glucose levels and homeostasis during pregnancy is critical.

Very few studies have examined if GDM can have a causative effect on fetus development on both the behavioral and molecular level. Appropriate mouse models would be necessary to determine such an effect. One mouse study examined how high-fat diet-induced gestational diabetes affects circulating immune factors in the maternal blood and cortical gene transcription in the fetus. They found dysregulated gene expression particularly in genes involved in the immune response and neurodevelopment^13^. However, that mouse model is not a specific model for gestational diabetes, considering the wide metabolic phenotype that is present in high-fat diet, and they did not determine if there was any behavioral effects on the offspring.

In the current study, we have developed a mouse model of GDM by injection of pregnant mice with the beta-cell toxin streptozotocin (STZ)^12^, followed by behavioral and molecular analysis of the offspring. We have discovered specific effects of GDM on behavior of offspring, neurodevelopment related genes in the frontal cortex, and specific transcriptome networks in the striatum. These findings support a causative role of GDM on altered neurodevelopment.

## Materials and methods

### Mice

C57BL/6 (C57) mice were used in the experiments. Behavioral tests were conducted at age of 8–10 weeks. Pups were weaned between postnatal 3–4 weeks. All procedures were approved by the Animal Ethics Committee of the Bar-Ilan University, Israel.

### Streptozotocin (STZ) administration

STZ was administered at day 1 of gestation (day of copulation plug) by a single intraperitoneal injection (120 mg/kg), while control mice received an injection of PBS vehicle.

### Glucose measurements

Blood glucose levels were measured daily using a FreeStyle Freedom Lite blood glucose meter (Abbott Diabetes Care Inc., CA, USA) by taking a single blood drop from the tip of the tail. For glucose measurement of offspring, blood samples were taken from offspring after decapitation.

### Behavioral experiments

The experiments were recorded with the Panasonic WV-CL930 camera and with the Ganz IR 50/50 Infrared panel. The recorded movement of the mice was analyzed by the Ethovision XT 10/11 (Noldus) software. All analysis was done blind to the treatment group.

At least 1 week before behavior tests, all mice were kept in a reversed cycle room, with food and water available ad libitum. For all animal behavioral experiments, we included animals from three experimental groups, the offspring of pbs-injected mothers (12 offspring), offspring of STZ-injected mothers that did not develop diabetes (STZ − D) (13 offspring), and the offspring of stz-injected mothers that developed diabetes (STZ + D) (10 offspring). A maximum of two male and two female mice were used from each litter, in order to include many litters in the analysis. Animals were excluded if they displayed signs of sickness, injuries (e.g., hunchback or wounds) and little locomotion. These criteria were pre-established.

### Open field (OF) locomotion and anxiety test

The OF test examines anxiety-like behavior in mice, and tests the conflict between the desire to explore a new open space and the desire to stay protected. The mouse is placed in the corner of a plastic square box where it moves freely for 10 min under ~120 LUX of light. We recorded the mouse for an additional 10 min in order to essay the grooming of the mouse.

### Marble burying test

The Marble burying test examines anxiety and obsessive compulsive disorder (OCD) behavior. The bases of the test is the observation that mice will bury objects in their bedding. Each mouse was placed individually into a Non-Glare Perspex (20 × 40 cm). Twenty green glass marbles (15 mm in diameter) were arranged in a 4 × 5 grid that covered 2/3 of the apparatus on top of 5 cm clean bedding. Each mouse was placed in the corner that did not contain the marbles and was given 30 min exploration period, after which the number of marbles buried, was counted. “Buried” was defined as 2/3 covered by bedding. Testing was performed under dim light (25 lux).

### Contextual and cued fear conditioning

Fear conditioning is a behavioral test in which the mice is learning to predict events. The mice learn the association between an aversive stimulus (electrical shock) and a particular neutral context (room, walls, smell) and neutral stimulus (tone). After the association is learned, the result can be an expression of fear response to the neutral stimulus or context. On habituation day the mouse was placed into a conditioning chamber (10.5 × 10.5 × 10.5 cm) and allowed to explore freely for 5 min. On the training day, each mouse was placed into a conditioning chamber and a tone (75 dB) was sounded as the conditioned stimulus for 30 s followed by a 2 s mild foot-shock (0.7 mA) as the unconditioned stimulus. One more tone-shock pair were given at 2 min intervals and the animal was returned to its home cage 30 s after the last pair. At 24 h after the conditioning session, the mice were placed back into the conditioning chamber for 5 min and their freezing behavior was measured in context. At 1 h after context testing, the mice were placed into a different, white Plexiglas chamber for 6.5 min and then the tone was turned on for 30 s. The freezing time was recorded.

### Rotarod

The test was conducted using an accelerating Rotarod (Med Associates, St. Albans, VT). The speed of the Rotarod was set to 40 rpm. The amount of time each mouse spent on the rod was measured. The latency to fall was recorded with a 300 s cutoff time. Latency to fall average was calculated from three trials per mouse.

### Social interaction test

The three-chamber paradigm was performed as previously described^14^. The three-chamber apparatus is a non-glare Perspex box (60 × 40 cm) with two gated walls that divide the apparatus to three chambers: left, center, and right (20 × 40 cm). The test mouse was placed in the middle chamber for habituation (5 min) when the gates are closed for both side chambers. During the sociability test, the gates are opened for a period of 10 min for the test mice to explore the whole arena, with one chamber hosting a novel mouse and the other chamber empty. Analysis of the time spent in each chamber is measured by EthoVision XT 10 (Noldus).

### Mouse brain microdissection

Immediately after decapitation, the brain was removed and placed into a 1-mm metal matrix (Stoelting, cat# 51380). The brain was sliced using the standard razor blades (GEM, 62-0165) into 2-mm slices that were quickly frozen on dry ice. The frontal cortex and striatum was punched using a 13-gauge microdissection needle on each hemisphere and stored in −80 °C. RNA extraction was performed using RNAeasy mini kit (Qiagen, Valencia, CA, USA).

### RNA sequencing and differential expression analysis

RNA-seq bioinformatics Adapters were trimmed from sequences using cutadapt tool (Martin 2011). Reads that were shorter than 40 nucleotides or that mapped to rRNA sequences (using Bowtie 1.0.0) were removed. TopHat (v2.0.10) was used to align the remaining reads to the mm10 genome. All libraries had at least 20 million uniquely aligned reads to the genome. Read counting to Refseq genes was done with HTseq-count (version 0.6.1p1) using the intersection_strict option, followed by differential expression analysis with DESeq2 using the options betaPrior FALSE, cooks Cutoff FALSE and independent Filtering FALSE (1.6.3). Raw *P* values were adjusted for multiple testing using the procedure of Benjamini and Hochberg. Gene ontology analysis on differentially expressed genes was performed using the Toppgenetool (https://toppgene.cchmc.org/). Supplementary Table 2 includes all genes, their fold change, and adjusted *P* values.

### WGCNA

In weighted gene correlation network analysis (WGCNA)^15^, Pearson’s correlations between gene expression data were used to build a signed network. A soft threshold of 12 was used for creating the adjacency matrix, chosen according to scale-free topology criterion. Next, Topological Overlap Matrix (TOM)-based dissimilarity measure was used for constructing dendogram of the network, and single modules, corresponding to dendogram branches, were defined using Dynamic Tree Cut algorithm. All modules, that essentially represent clusters of highly interconnected genes, were given a color. Each module must have a minimum of 30 genes in the module. The module eigengene (ME) was calculated for every sample in each module. ME values correspond to the first principal component of the particular module and can be considered as a representative of expression profiles of genes from that module. To evaluate module-trait associations, the ME values of samples from each module were correlated to applied treatments. Also, WGCNA defines the module membership (MM) for each gene, which represents the correlation between its expression levels and the ME values across all samples. For those modules that showed significant correlation with maternal diabetes, additionally, we evaluated association between MM and gene significance (correlation between expression of that gene and diabetes state). Supplementary Table 3 includes all genes, the module association, and their MM for each module.

### Real-time PCR analysis

RNA was reverse transcribed to cDNA using the High Capacity RNA to cDNA kit (Applied Biosystems, Foster City, CA). cDNA was then analyzed by quantitative RT-PCR. Real-Time PCR on microbial DNA was performed using primers found in Supplementary Table 1. Real-time PCR was performed using FastStart Universal SYBR Green Master (Rox) (Roche) and analyzed with ViiA™7 Real-Time PCR System (Applied Biosystems).

Real-time PCR was performed using FastStart Universal SYBR Green Master (Rox) (Roche) and analyzed with ViiA™7 Real-Time PCR System (Applied biosystems).

### Immunofluorescence staining

Mice pancreatic tissue was fixed in 4% formaldehyde (PFA) over night at 4 °C, washed with PBS, followed by overnight incubation in 30% sucrose at 4 °C. Next, the tissue was embedded Optimal Cutting Temperature and was frozen in a mixture of dry ice and 2-methylbutane. The frozen blocks were mounted on Leica cryostat and cryosections of 5 μm thick were prepared. Cryosections were air-dried and washed three times with PBS followed by incubation with 0.3% Triton in PBS for 15 min. After washing with PBS for 15 min, slides were incubated in blocking solution with 5% normal donkey serum for 45 min at room temperature. All cryosections were incubated with antisera specific for insulin, glucagon, and somatostatin. Secondary antibodies have been labeled with Alexa Fluor 647, Alexa Fluor 488, and Rhodamine Red. Immunofluorescence images were taken with the Zeiss Axio Scan.Z1 Slide scanner through a 40× objective.

### Statistical analysis

All statistical analysis data were analyzed by either two tail independent *T* Test or two way ANOVA, when appropriate. The Levene’s test was used to test for equality of variance between groups. Whenever the variance was unequal, Welch’s *T* Test was used to test for significance. *p* values less than 0.05 were considered significant. The data are presented as mean ± standard error of the mean. Statistical analysis of the staining was done by using SigmaPlot.

## Results

### Induction of gestational diabetes

**Fig. 1:**
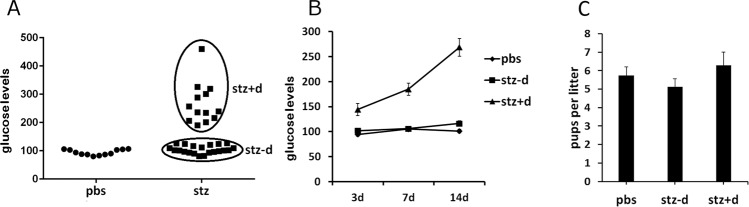
STZ-induced gestational diabetes. **A** Blood Glucose levels at 2 weeks of pregnancy (14 days) in pregnant female mice that were injected with STZ at E0.5. STZ-treated mice either develop gestational diabetes by 2 weeks of age (STZ + D) or show very little increase in blood glucose levels (STZ − D), which are similar to pbs-streated mice. **B** Blood glucose level analysis of pregnant mothers at 3 days, 7 days, and 14 days post conception. Differences between STZ + D and STZ − D groups are already present at 3 days of pregnancy, but reach hyperglycemia only at 14 days of pregnancy. **C** Number of pups per litter in each experimental group. *n* = 8 litters per experimental group. Data are presented as mean ± standard error of the mean.

In order to induce gestational diabetes, without influencing pancreatic development in the offspring, STZ was injected i.p. to pregnant females at E0.5. At this early stage of fetal development, the pancreas is not formed (the embryonic pancreas is starting to form at E9.5)^16–18^. Maternal blood glucose levels were then determined at three days, 1 week and 2 weeks after initiation of pregnancy. By two weeks of pregnancy, a subset of dams displayed diabetes (mg/dL>200), while a subset showed very little increase in blood glucose levels (Fig. 1A). Interestingly, the effect of STZ was binary, with either a strong effect on blood glucose levels, or very little effect. Further analaysis determined that the diabetic mice displayed an increase in blood glucose levels as early as 3 days after injection, but the levels only reached hyperglycemia by 2 weeks of pregnancy (Fig. 1B). Therefore, we performed all our experimentation on three groups: PBS treated, STZ treated with no development of diabetes (STZ − D), and STZ treated with development of diabetes (STZ + D). While it is well established that STZ induces diabetes in only a subset of mice, and most studies disregard the animals that do not develop diabetes, we kept that group as an additional control group in our analysis. We further verified that there are no differences in litter size between the three experimental groups (Fig. 1C), and the expected equal male vs. female ratio of pups among the offspring was observed in all experimental groups.

### Effect of STZ treatment to mothers on morphology and function of offspring pancreas

**Fig. 2:**
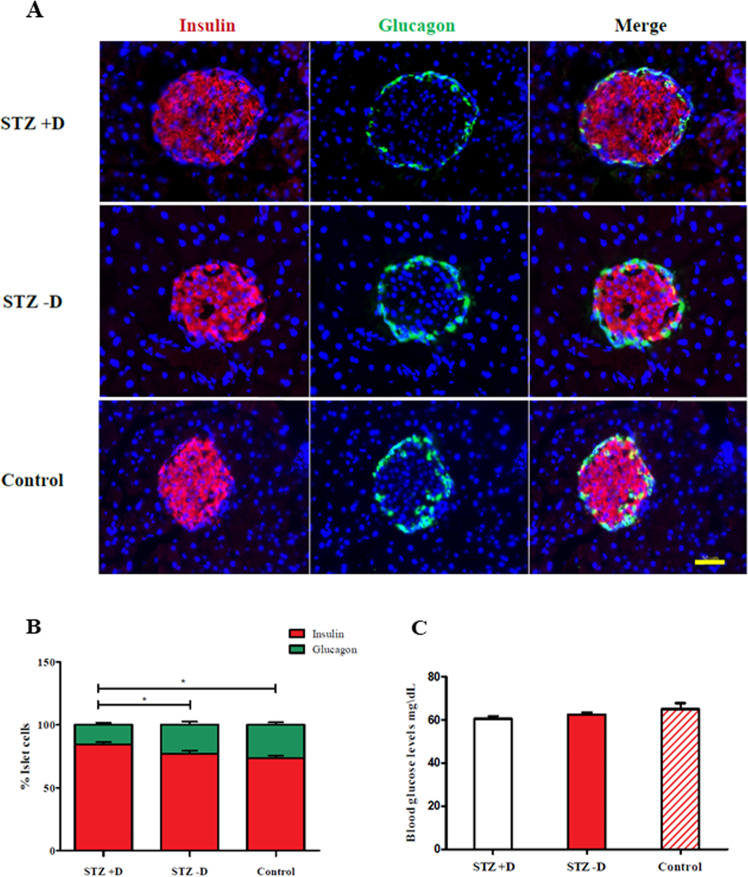
Effects of STZ treatment on pancreas of offspring. **A** Immunofluorescence staining of the STZ + D, STZ − D and Control offspring’s pancreata. Insulin in red, Glucagon in green and DAPI in blue. scale bar = 50 µm. **B** Comparing the percentage of islet cells in all groups. *n* = 30 STZ + D, *n* = 22 STZ − D and *n* = 16 control. *p < 0.05. **C** Blood glucose levels of all groups’ offspring. *n* = 23 STZ + D, *n* = 22 STZ − D and *n* = 22 control.

To examine if the STZ treatment in pregnant mice affected the development of insulin-producing cells in the offspring, we performed immunofluorescence staining on offspring pancreases. Staining for insulin and glucagon reveals intact Islets of Langerhans in the pancreas of all experimental groups (Fig. 2A), with insulin positive cells. Of interest, there was a subtle, but significance increase in relative levels of insulin positive cells in the STZ + D group (Fig. 2B). This may be explained by recent findings that a glucose-rich environment during development can stimulate the genesis of beta cells^19^. In addition, we tested blood glucose levels in PBS, STZ − D, and STZ + D offspring, and found no significant differences in glucose levels between the experimental groups (Fig. 2C). Therefore, STZ treatment did not induce decreases in beta-cell levels and had no significant effect on blood glucose levels in the adult offspring.

### Effects of gestational diabetes on behavior in adult offspring

**Fig. 3:**
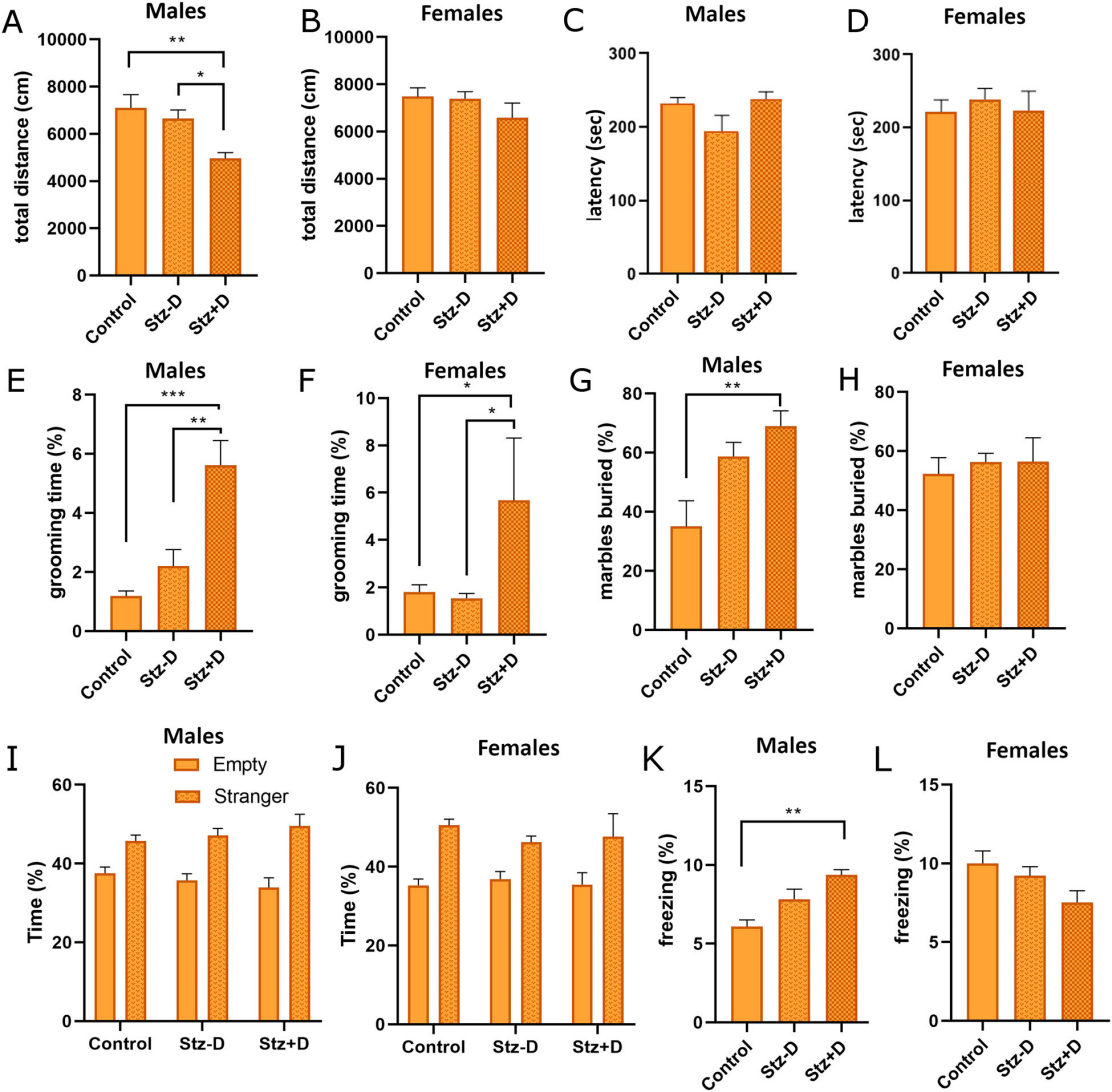
Effects of Gestational diabetes on behavior. Total distance traveled in the open field test in male (**A**) and female (**B**) mice shows less distance traveled specifically in the male STZ + D mice. Rotarod tests shows no differences in locomotor abilities in all experimental groups in both male (**C**) and female (**D**) mice. Grooming time was calculated in all experimental groups during a 10-minute period in the open field maze. There is a significant increase in grooming time in the STZ + D group of both male (**E**) and female (**F**) mice. Mice were subjected to a 30-min period in open chamber with 20 marbles. Number of buried marbles were counted. STZ + D group showed significant increase in buried marbles, compared to control group in the male mice (**G**). No differences are seen in the female mice (**H**). Data are presented as mean ± standard error of the mean. **I**, **J** Mice were subjected to the three chambered social interaction test. All experimental groups displayed normal preference toward stranger mice compared to an empty chamber in both male (**I**) and female (**J**) mice. **K**, **L** Cue Fear conditioning test was carried out in all experimental groups. In male mice (**K**), STZ + D mice display significantly more freezing, compared to control mice. There were no differences between groups in female mice. Data are presented as mean ± standard error of the mean. One way Anova. *p < 0.05 *n* = 10–13.

To test the effect of maternal diabetes on anxiety and locomotor behavior, we tested the adult offspring in the OF test. Among the male offspring, the STZ + D group moved significantly less in the OF chamber, compared to either the control group or STZ − D group (Fig. 3A). In contrast, among the female offspring, there was no difference between the groups in total distance moved or time in center (Fig. 3B). In order to determine if the decrease in distance traveled is due to underlying motor impairments, we performed the rotarod test. No differences were found between the groups, indicating that there is no underlying motor impairments in the STZ + D group (Fig. 3C, D).

In order to better examine behavior in the OF, we determined grooming time for each mouse. Interestingly, STZ + D mice displayed more grooming behavior for both males and females offspring (Fig. 3E, F). In addition, when including mice form all experimental groups, we found a positive correlation between maternal glucose levels and grooming behavior (Pearson correlation *r* = 0.58 *p* = 0.00026). These findings indicate that males have increased repetitive behavior, which may also partially explain the decrease in locomotion time among these animals.

In order to further verify if there are changes in repetitive behavior, we performed the marble burying test. Male pups of diabetic mothers (STZ + D) buried significantly more marbles than the control group (Fig. 3G). In addition, there was a positive correlation between maternal glucose levels and marble burying behavior among the male offspring (Pearson correlation *r* = 0.38 *p* = 0.024). However, there were no differences between groups in the female mice (Fig. 3H). These results indicate a consistent increased in repetitive behavior of STZ + D male offspring.

The social behavior of the mice was determined in three-chamber social interaction maze, by calculating the time the tested mouse spent with a stranger mouse, compared with an empty chamber (sociability test). In sociability test, all mice, males and females, preferred the chamber with the stranger mice over the empty chamber (Fig. 3I, J). Therefore, STZ + D mice displayed no deficits in the three-chamber social interaction test.

In the fear conditioning paradigm, male STZ + D mice displayed increased freezing in the cue test (Fig. 3K). Female STZ + D mice displayed no significant differences in freezing (Fig. 3L). In the context test, there was no difference between groups (Supplementary Fig. S1). In addition, there were no differences in freezing among experimental groups during the habituation period, which suggests that the differences are specific to fear memory, and not related to differences in the locomotor function (Supplementary Fig. S2).

### RNA-seq analysis of the frontal cortex and striatum from mice of diabetic and non-diabetic mothers reveals changes in gene expression

**Fig. 4:**
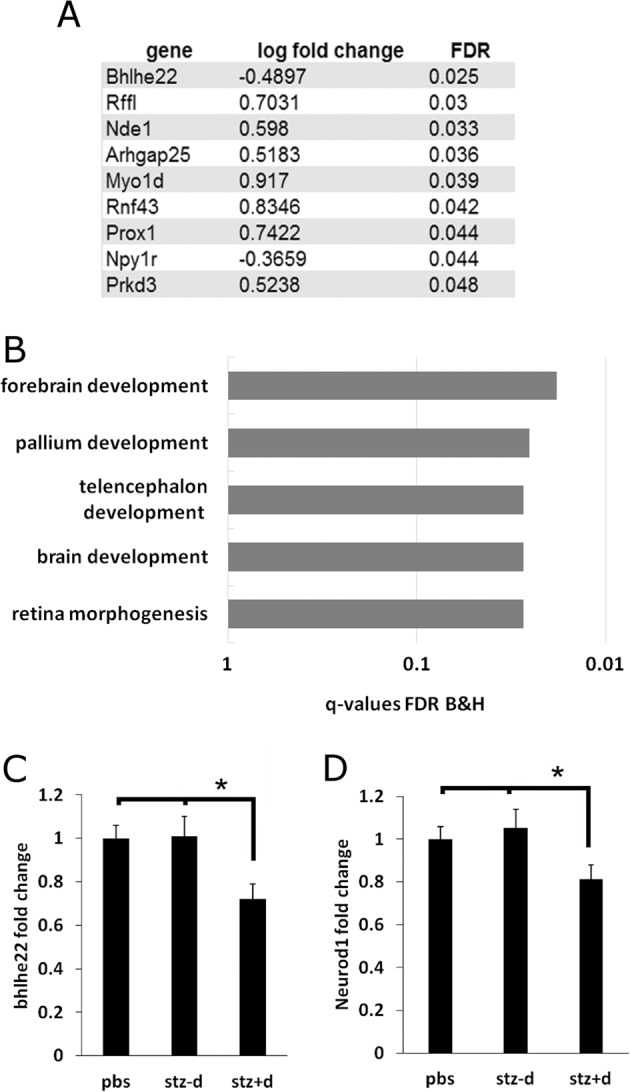
Effects of gestational diabetes on cortical gene transcription. RNA-seq analysis was performed on frontal cortex and striatum samples from all experimental groups. **A** List of genes that were differentially expressed in the STZ + D frontal cortex. **B** Gene ontology analysis of the differentially expressed genes from the frontal cortex shows enrichment of genes involved in forebrain development. **C**, **D** Real-Time PCR analysis of genes bhlhe22 and Neurod1 in all three experimental groups. Data are presented as mean ± standard error of the mean. One way Anova. *p < 0.05 *n* = 7 per experimental group.

To determine a possible molecular mechanism that may be involved in the behavior changes that we observed, we performed whole-genome RNA sequencing on frontal cortex and striatal tissue from male offspring of the three experimental groups. Differential expression analysis determined that nine genes were differentially expressed between the STZ − D and STZ + D groups in the frontal cortex (Fig. 4A, Supplementary Table 2). Gene ontology analysis revealed that the list is enriched for genes involved in forebrain development (Fig. 4B). These forebrain development related genes include Myo1d, Nde1, Bhlhe22, and Prox1. Of additional interest, the gene Neurod1, which is highly implicated in neuron development and in diabetes, had a strong tendency for differential expression (FDR = 0.058). We further used real-time PCR to validate the decreases in bhlhe22, the most significantly dysregulated gene, and neurod1 (Fig. 4C). Bhlhe22 is a DNA-binding protein that has been shown in multiple studies to be involved in proper development of cortical area identity^20,21^ and regulates development of neuronal circuits^22^. We found that these genes were decreased specifically in the STZ + D group, compared to both the PBS and STZ − D group. There were no genes that were differentially expressed between the control and STZ − D group, verifying that STZ treatment alone does not affect adulthood gene transcription. In differential analysis of the striatal transcriptome, there were no significantly dysregulated genes among the experimental groups.

**Fig. 5:**
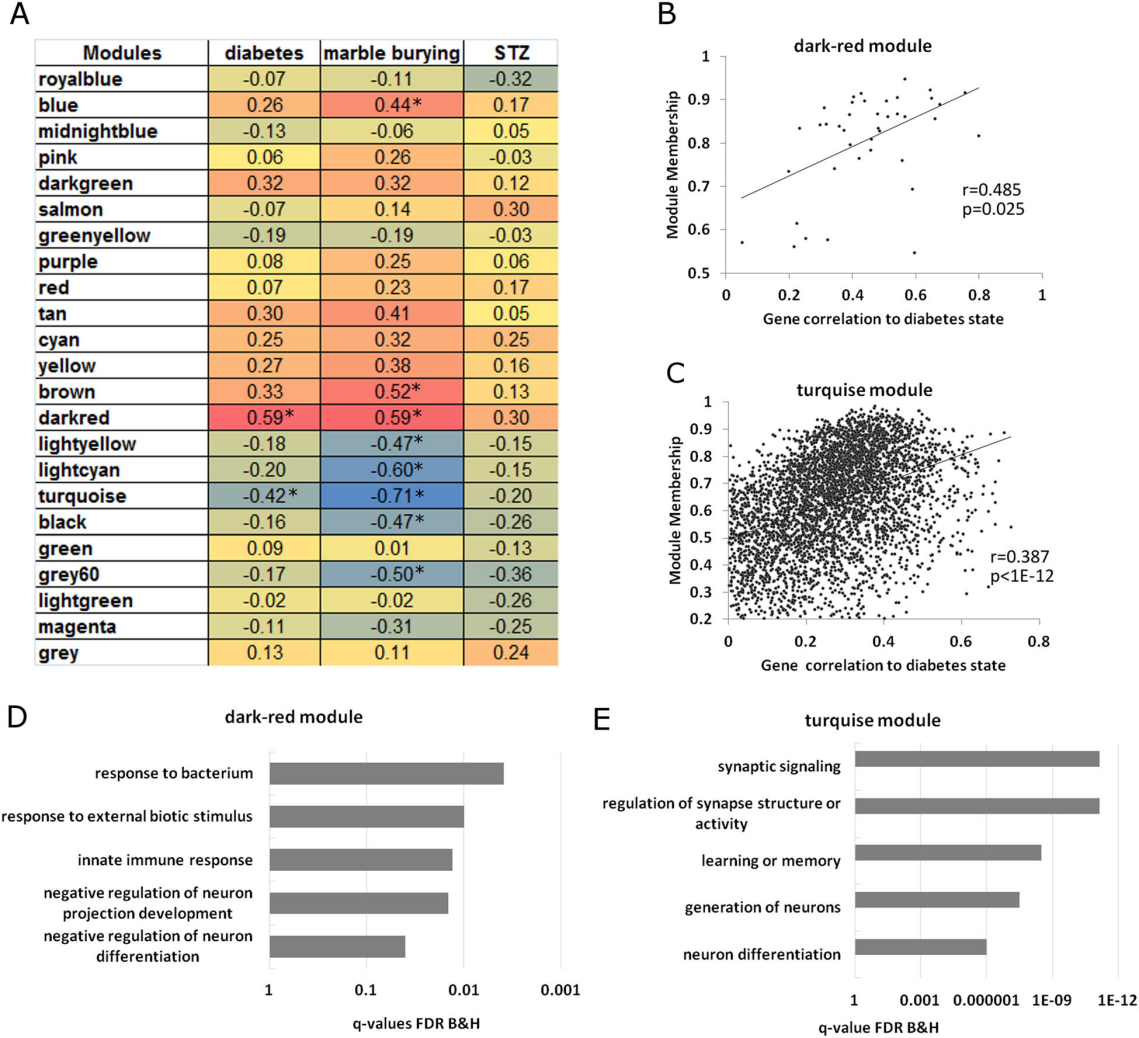
Effects of gestational diabetes on striatal gene transcription. **A** WGCNA analysis of striatal RNA-seq data. Modules were correlated to maternal diabetes (STZ + D group), amount of marbles buried, and STZ treatment. **B**, **C** Scatter plots indicate the correlation (Pearson’s *r* correlation coefficient) and its significance (*p* value) between module membership and gene significance (gene correlation to diabetes state). The positive correlation gives further evidence that genes in these modules are associated with the state of gestational diabetes in the mother. **D**, **E** Gene ontology analysis of genes in the dark-red and turquoise modules.

While differential expression analysis is effective to identify individual genes that are differentially expressed in the diabetic group, WGCNA is particularly effective in identifying groups of genes with common biological mechanisms that are correlated with the diabetic phenotype and are correlated with specific behaviors. WGCNA is a network-based method that discovers coexpressed genes among the samples (modules). We can then test if the average expression of these coexpressed genes (eigengene) is correlated with diabetes or behavioral phenotypes. In the striatum transcriptome data, we observed a significant correlation between “dark-red” module and the diabetic phenotype (Fig. 5A). In addition, there was a slight correlation between the turquoise module and the diabetic phenotype (*p* = 0.055). In addition, the dark-red module was positively correlated with number of marbles buried, while the turquoise module was very strongly negatively correlated with the marbles buried phenotype. No phenotypes were correlated with STZ treatment, verifying that the effects were specific for the diabetes and behavioral phenotypes. We verified the relationship between these two modules and the diabetes phenotype by showing that MM of each gene is strongly correlated to that gene’s gene significance (correlation to the diabetes genotype) (Fig. 5B, C). In other words, genes that are strongly correlated with this module are strongly correlated with the diabetes phenotype.

Gene ontology analysis was performed to understand the biological significance of the genes in each module. The dark-red module is enriched for genes involved in immune functions as well as in genes involved in negative regulation of neuron differentiation and projection development (Fig. 5D). These neuron-related genes include CARM1, CDK5R1, HLA-A, and B2M. The Turquoise module consists of 3953 genes and is enriched for genes involved in synaptic processes such as “synaptic signaling” as well as in genes involved in neuron generation and differentiation (Fig. 5E). Therefore, the WGCNA method has uncovered a significant increase of genes involved in the downregulation of neuron development in the striatum and a decrease in genes involved in positive neuron development.

## Discussion

The current study has determined a dysregulation in behavior and changes in the cortical and striatal transcriptome in the offspring of a novel mouse model of gestational diabetes. The most robust behavioral changes were in indexes of repetitive behaviors, where GDM offspring displayed increased repetitive behaviors. Behavioral dysregulation was more consistent in male offspring than female offspring. This is consistent with the male bias of autism prevalence in humans. Male mice also displayed decreased distance traveled in the OF, and index of exploratory activity, with no changes in rotarod, which suggests that this is not an issue of motor function. While the decreased activity in the OF test may be partly explained by increased grooming behavior, this still does not fully explain the dysregulation in activity. It is possible that GDM offspring are also displaying less exploratory behavior as a result of increased fear. The increase in fear conditioning in these mice further suggests that this may be a possible interpretation of the results. GDM offspring did not show any dysregulation in the classic three-chambered social ability paradigm, suggesting that there are not major effects on social function. However, in recent years, more robust tests of social behavior are being developed. We cannot rule out that more subtle social behaviors may be effected, which we were not able to detect in the three-chambered test.

In order to understand GDM-induced dysregulation in the brain, we determined the cortical and striatal transcriptome of male mice in all three experimental groups. These two brain regions were chosen because they both have previously been implicated in the regulation of repetitive and motor behaviors^23,24^. Nine genes were differentially expressed in the frontal cortex, and these genes were enriched for the function of forebrain development. Some of these genes have particular roles in cortical development, including bhlhe22 and Nde1. Nde1 has been implicated in the development and regulation of size of the mammalian cortex^25,26^. Bhlh22 is a DNA-binding protein which has previously been found to be central in establishment of neuronal circuits^27–29^. Knockout of bhlhe22 induces disorganization and aberrant gene expression in cortical areas 2–5^27^. A separate study of the bhlhe22 knockout mouse found a dysregulation of neuronal circuits during development, including a behavioral phenotype of self injury by overgrooming^28^. This is reminiscent to our finding of overgrooming in the GDM offspring. Therefore, decreased bhlhe22 in the cortex may be partly responsible for this behavior.

In the striatal transcriptome data, there is one module (turquoise), that negatively correlates both with GDM and with the behavioral phenotype of buried marbles. This module in enriched for genes involved in synaptic signaling, suggesting that signaling processes in the striatum are partially responsible for the marble burying phenotype. This correlates with the well known role of the striatum in motor and repetitive behaviors^30^. One limitation of our gene expression analysis is that it was performed only in male offspring. While the behavioral phenotype was less prominent in the female mice, they also showed increased grooming behavior, and it would be of interest to understand sex-specific effects of GDM on gene transcription. In total, dysregulation of forebrain development-related genes and striatial synaptic-signaling genes further suggests a link between maternal diabetes and dysregulation of neuronal development.

An interesting phenomenon observed is the relative beta-cell mass in STZ + offspring. Different reports have indicated that beta-cell function was hampered in offspring of diabetic mothers in human and rodents^31,32^. However, beta-cell mass was not calculated in these reports. We did not see these observations. While blood glucose levels were similar in all pups, offspring of STZ + mothers displayed a small, yet statistically significant increase in pancreatic beta-cell area. This comes in correlation of similar observations where embryos displayed increase beta-cell mass with the increase of the fetal environmental glucose levels^19^.

While our study is the first to show the effects of diabetes induced after initiation of pregnancy (gestational diabetes), previous studies have looked into effects of general maternal diabetes on offspring behavior. Money et al. used high-fat diet to induce a diabetes-like phenotype in pregnant females, and then looked at the cortical transcriptome at age E12.5 of the offspring^13^. They found dysregulation of genes involved both in neurodevelopment and the immune response. This study gave some initial indication of the effects of maternal diabetes on offspring development. However, there was no determination of behavioral effects in the offspring. In addition, the high-fat model induces a wide range of phenotypes, which is not specific to diabetes, including increase in fat mass, decreases in lean body mass, and other metabolic effects, which could all affect neurodevelopment. In a more recent study, Wang et al. induced maternal diabetes by STZ administration, followed by initiation of pregnancy^33^. They found that offspring displayed social behavior deficits that could be attenuated by amygdalar overexpression of SOD2 or treatment with antioxidants. In our study, there is no effect on social behaviors, but rather an effect on repetitive behaviors. An important difference in our studies is that they verified a diabetic phenotype before initiation of pregnancy, while in our model, glucose reaches diabetic levels between E7 and E14 (more closely resembling gestational diabetes). Therefore, different behavioral deficiencies may be dependent on the presence of maternal hyperglycemia at different developmental time points.

The GDM model presented in this study has clear advantages over the previous models. However, there are still some limitations that should be considered. First, STZ-induced destruction of pancreatic beta-cells is a model of type-1 diabetes, while GDM in humans does not include beta-cells destruction. In addition, hyperglycemia is already present at day 3 in these mice, before organogenesis, while GDM in humans usually occurs after organogenesis. However, the most significant and robust changes in glucose levels in our model was at day 14, well after the establishment of organogenesis. One other consideration for the present study is the possible role of maternal care in the behavior of offspring. In other words, further research is necessary to fully determine how much of the behavioral phenotype is due to prenatal effects of GDM, or if there could be influence of altered maternal care among diabetic mothers. With the above limitations in mind, the GDM model used in our manuscript is still the only model, to our knowledge, to induce maternal diabetes in mice with no preexisting condition before the intitiation of pregnancy, and is therefore a significant step forward in our ability to model GDM in mice.

This study gives evidence that gestational diabetes may affect an autism related behavior, particularly repetitive behaviors, and can be involved in dysregulated neurodevelopment at the level of gene expression. Future studies should help to understand the functional connection between the maternal diabetic phenotype and dysregulated brain transcriptome in the offspring.

## Supplementary information

Supplementary Figures

Supplementary Table 1

Supplementary Table 2

Supplementary Table 3
