# Supplementary material for: Gestational diabetes induces behavioral and brain gene transcription dysregulation in adult offspring
Source: Transl Psychiatry. 2020 Nov 25;10:412. doi: 10.1038/s41398-020-01096-7 (PMC7688640; doi:10.1038/s41398-020-01096-7)
Supplement: Supplementary file 1 — Supplementary Figures [file 41398_2020_1096_MOESM1_ESM.docx]

**Gestational Diabetes induces behavioral and brain gene transcription dysregulation in adult offspring**

Supplemental Figures


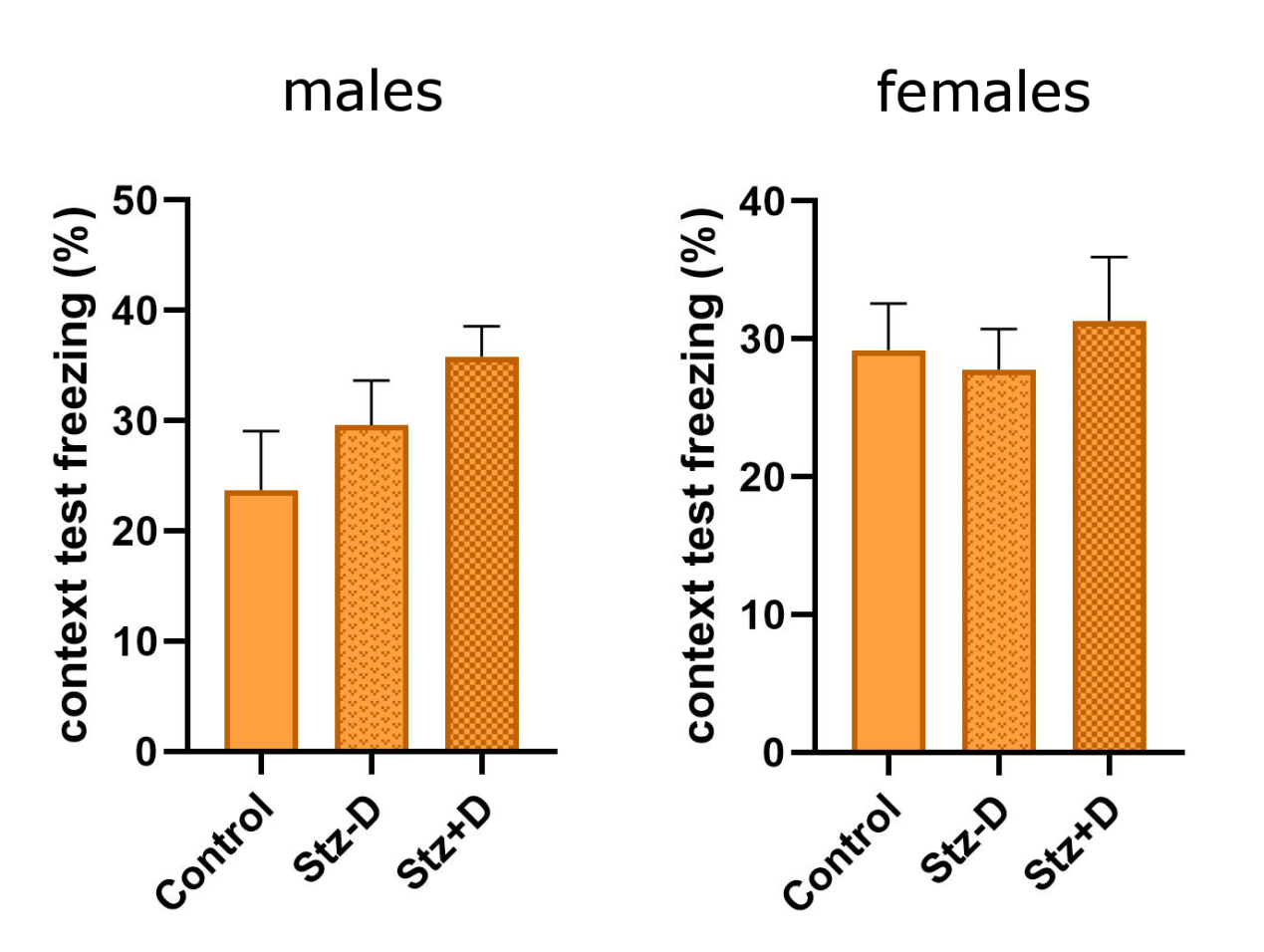


**Supplementary Figure S1 | Contextual Fear Conditioning Analysis in all three experimental groups.** Amount Time freezing in contextual fear conditioning analysis in both males (a) and females (b). There are no differences in contextual fear conditioning in all three groups. One Way Anova. n=10-13.


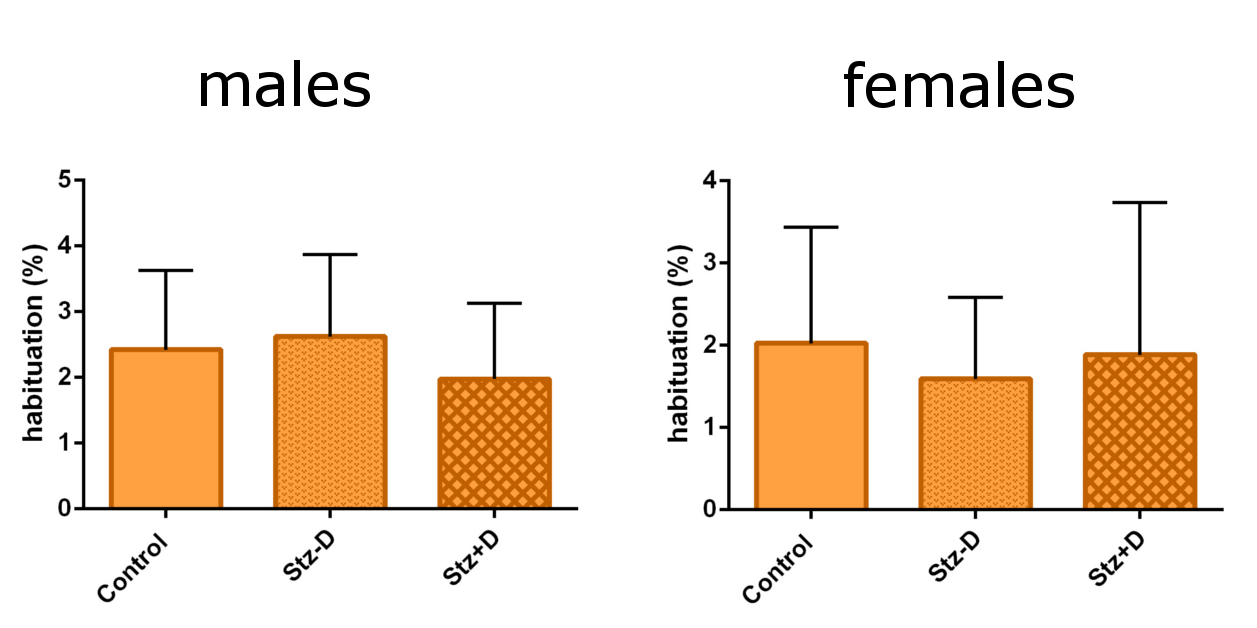


**Supplementary Figure S2 | Freezing time during habituation the day before fear conditioning trials** Amount Time freezing in habituation one day before fear conditioning analysis in both males (a) and females (b). There are no differences in freezing during habituation in all three groups. One Way Anova. n=10-13.
